# Supplementary material for: Methyl Jasmonate Enhances the Resistance of Populus alba var. pyramidalis Against Anoplophora glabripennis (Coleoptera: Cerambycidae)
Source: Insects. 2025 Feb 3;16(2):153. doi: 10.3390/insects16020153 (PMC11855635; doi:10.3390/insects16020153)
Supplement: Supplementary file 1 [file insects-16-00153-s001.zip › insects-3389087-supplementary.pdf]

Supplementary Materials:

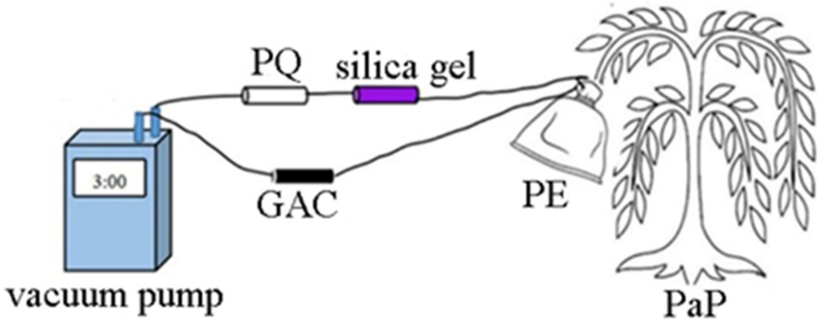

Figure S1. Volatiles collection equipment in the field experiment

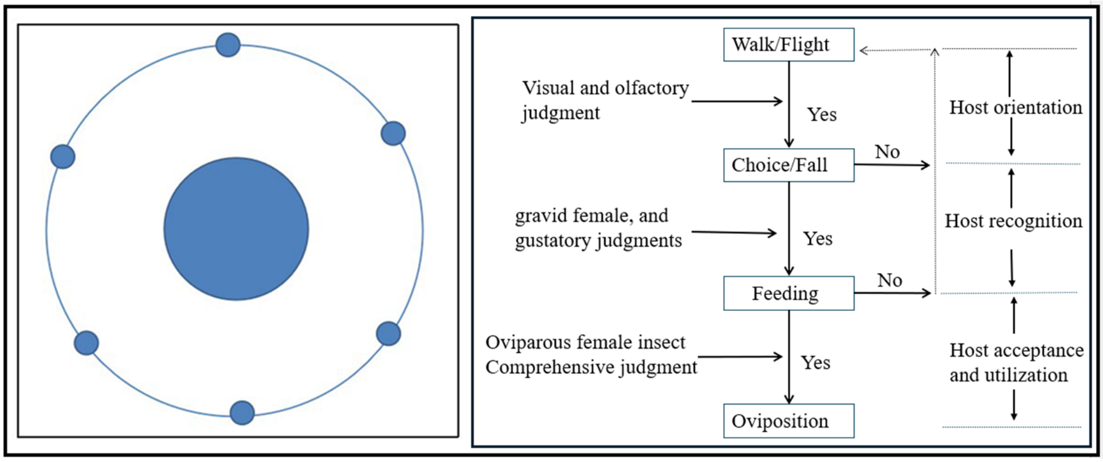

Figure S2. Test scheme and interaction of ALB and host tree

Note: the ALB adults were released at the centre of the tested arena, and six points around the circle represent the treatments with different concentration of MeJA and control plant.

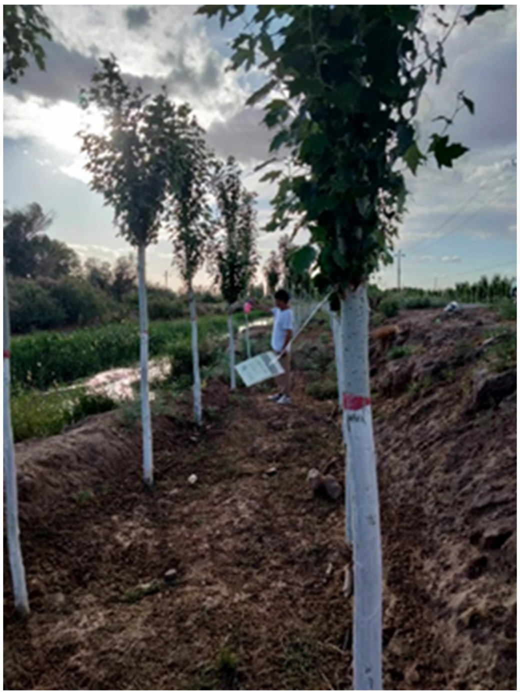

Figure S3. Field spray of MeJA on PaP

Note: Two meters between different tested trees in the field.

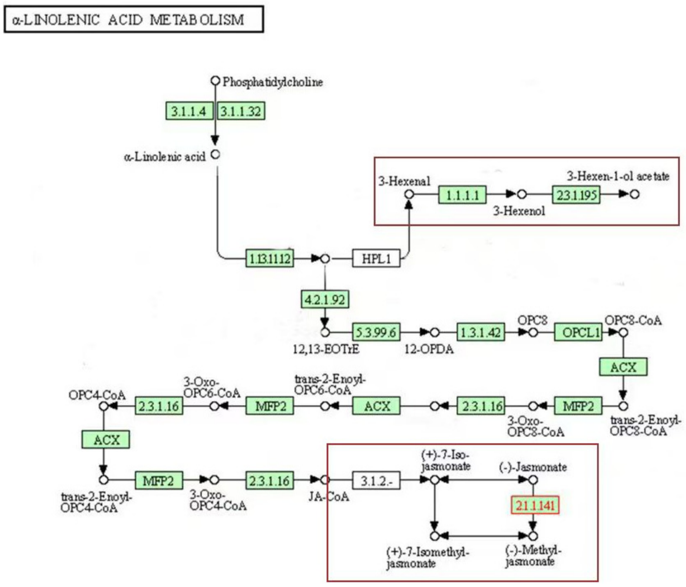

Figure S4. **Z3HA, Z3H and the metabolic pathways of SA, JA, MeSA and MeJA**

Note: This map is from KEGG (https://www.genome.jp/kegg/;00592 pathway results; URL, accessed on July 18, 2022) .

Tables

Table S1. Volatile compounds emitted from *Populus alba.* var. *pyramidalis*

No	Peak time (min)	Molecular formula	Chemical name	CAS No	Content (%)
1	4.445	$C_6H_{12}O$	Hexanal	66-25-1	0.44
2	5.541	C_8H_{10}	Ethylbenzene	100-41-4	0.28
3	5.758	C_8H_{10}	P-XYLENE	106-42-3	0.82
4	6.180	$C_6H_{10}O$	3-hexen-1-al	4440-65-7	0.58
5	8.234	C_6H_{10}	Trans-2-Hexenal	6728-26-3	3.08
6	8.355	C_9H_{12}	4-Ethyltoluene	622-96-8	0.32
7	8.464	$C_7H_{12}O_2$	(2Z)-pent-2-en-1-yl acetate	42125-10-0	0.25
8	9.926	$C_8H_{16}O_2$	Hexyl acetate	142-92-7	0.73
9	9.982	C_9H_{12}	Mesitylene	108-67-8	0.52
10	11.326	$C_8H_{14}O_2$	(Z)-3-Hexen-1-yl acetate	3681-71-8	23.55
11	11.679	$C_8H_{14}O_2$	(*Z*)-hex-2-enyl acetate	56922-75-9	1.72
12	12.188	$C_7H_{14}O_2$	hexyl formate	629-33-4	6.30
13	13.689	$C_6H_{12}O$	(*Z*)-3-Hexenol	928-96-1	16.03
14	15.229	$C_{10}H_{18}O_2$	Cis-3-Hexenyl Butyrate	16491-36-4	4.06
15	15.507	$C_{11}H_{20}O_2$	Cis-3-Hexenyl 2-Methylbutanoate	53398-85-9	6.88
16	16.068	$C_{13}H_{22}O$	2,6,10,10-Tetramethyl-1-oxaspiro[4.5]dec-6-ene	36431-72-8	0.29
17	16.777	C_7H_6O	Benzaldehyde	100-52-7	0.46
18	18.562	$C_{15}H_{24}$	*β*-Caryophyllene	87-44-5	2.07
19	20.693	$C_7H_6O_2$	Salicylaldehyde	90-02-8	0.84
20	21.437	$C_9H_{10}O$	3-Ethylbenzaldehyde	34246-54-3	2.77
21	22.977	$C_8H_8O_3$	Methyl salicylate	119-36-8	0.59
22	24.256	$C_{10}H_{12}O$	1-(3-ethylphenyl)ethenone	22699-70-3	8.61
23	25.065	$C_{10}H_{12}O$	4-Ethylacetophenone	937-30-4	11.33
24	27.588	$C_{10}H_{14}O$	4-Isopropylbenzyl Alcohol	536-60-7	2.90
25	27.997	$C_{10}H_{14}O$	Thymol	89-83-8	4.62
26	28.606	$C_{11}H_{14}O$	O-methyleugenol	93-15-2	0.89
27	34.293	$C_{10}H_{10}O_2$	1,4-diacetylbenzene	1009-61-6	0.70
28	34.871	$C_{11}H_{14}O$	4'-Isopropylacetophenone	645-13-6	0.81

Table S2: Class, analyte, linear range, regression equations, correlation coefficient and weight of the phytohormones

Phytohormones	Substance name	Linear range (ng/mL)	Calibration curve	Correlation coefficient (r)
JA	JA	0.1-50	$y = 1.9\,x + 0.0043$	$r = 0.9952$
	MeJA	0.5-50	$y = 1.29\,x + 0.0132$	$r = 0.9913$
SA	SA	0.5-200	$y = 1.4\,x + 0.0182$	$r = 0.9992$
	MESA	0.1-200	$y = 26.3\,x + 0.00777$	$r = 0.9975$

Table S3: The original data for quantities of different plant hormones

Sample Number	Sample weight (g)	Sample volume（mL）	SA	JA	MeSA	MeJA	SA	JA	MeSA	MeJA
			ng/mL				ng/g			
Infested P1	1.59	0.4	71.30	2.94	9.21	8.03	17.90	0.74	2.31	2.02
Infested P2	1.58	0.4	68.80	2.77	8.20	8.39	17.50	0.70	2.08	2.13
Infested P3	1.50	0.4	67.50	1.96	7.45	6.80	18.00	0.52	1.99	1.81
Healthy P4	1.53	0.4	109.00	1.22	9.44	2.74	28.50	0.32	2.47	0.72
Healthy P5	1.51	0.4	108.00	1.29	9.54	2.15	28.60	0.34	2.53	0.57
Healthy P6	1.50	0.4	106.0	1.03	9.24	2.65	28.30	0.27	2.46	0.71
CK	0	0.4	0	0	0	0				
